# Synthesis and characterization of *d*_5_‐barbarin for use in barbarin‐related research

**DOI:** 10.1002/dta.3357

**Published:** 2022-08-23

**Authors:** Sucheta Kudrimoti, Jacob Machin, Adedamola S. Arojojoye, Samuel G. Awuah, Rodney Eisenberg, Clara Fenger, George Maylin, Andreas F. Lehner, Thomas Tobin

**Affiliations:** ^1^ The Department of Veterinary Science and the Maxwell H. Gluck Equine Research Center and the Department of Toxicology and Cancer Biology University of Kentucky Lexington Kentucky USA; ^2^ Department of Chemistry University of Kentucky Lexington Kentucky USA; ^3^ Center for Pharmaceutical Research and Innovation, College of Pharmacy and Department of Pharmaceutical Sciences, College of Pharmacy University of Kentucky Lexington Kentucky USA; ^4^ Frontier BioPharm, LLC Richmond Kentucky USA; ^5^ Equine Integrated Medicine Georgetown Kentucky USA; ^6^ New York Drug Testing and Research Program Ithaca New York USA; ^7^ Veterinary Diagnostic Lab Section of Toxicology Michigan State University Lansing Michigan USA

**Keywords:** aminorex, *Barbarea vulgaris*, *d*_5_‐barbarin, equine forensic science, internal standard

## Abstract

Based on structural similarities and equine administration experiments, Barbarin, 5‐phenyl‐2‐oxazolidinethione from *Brassicaceae* plants, is a possible source of equine urinary identifications of aminorex, (*R,S*)‐5‐phenyl‐4,5‐dihydro‐1,3‐oxazol‐2‐amine, an amphetamine‐related US Drug Enforcement Administration (DEA) controlled substance considered illegal in sport horses. We now report the synthesis and certification of *d*_5_‐barbarin to facilitate research on the relationship between plant barbarin and such aminorex identifications. *D*_5_‐barbarin synthesis commenced with production of *d*_5_‐2‐oxo‐2‐phenylacetaldehyde oxime (*d*_5_‐oxime) from *d*_5_‐acetophenone via butylnitrite in an ethoxide/ethanol solution. This *d*_5_‐oxime was then reduced with lithium aluminum hydride (LiAlH_4_) to produce the corresponding *d*_5_‐2‐amino‐1‐phenylethan‐1‐ol (*d*_5_ *‐*phenylethanolamine). Final ring closure of the *d*_5_‐phenylethanolamine was performed by the addition of carbon disulfide (CS_2_) with pyridine. The reaction product was purified by recrystallization and presented as a stable white crystalline powder. Proton NMR spectroscopy revealed a triplet at 5.88 ppm for one proton, a double doublet at 3.71 ppm for one proton, and double doublet at 4.11 ppm for one proton, confirming *d*_5_‐barbarin as the product. Further characterization by high resolution mass spectrometry supports the successful synthesis of *d*_5_‐barbarin. Purity of the recrystallized product was ascertained by High Performance Liquid Chromatography (HPLC) to be greater than 98%. Together, we have developed the synthesis and full characterization of *d*_5_‐barbarin for use as an internal standard in barbarin‐related and equine forensic research.

## Graphical abstract

We now report the synthesis of *d*_5_‐barbarin for use in research concerning the role of barbarin in glucobarbarin‐related identifications of aminorex in experimental and postcompetitive event urine samples from horses fed plants containing glucobarbarin. We also propose a synthetic pathway for aminorex formation from glucobarbarin.

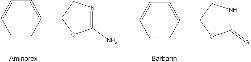

## 1 INTRODUCTION

**FIGURE 1:**
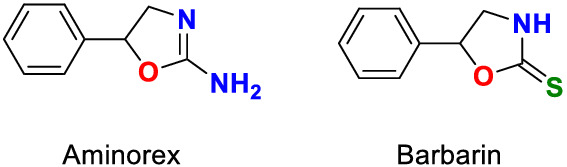
Structures of aminorex and barbarin. Aminorex, (*R*,*S*)‐5‐phenyl‐4,5‐dihydro‐1,3‐oxazol‐2‐amine, molar mass, 162.19 g/mol (left), and barbarin, 5‐phenyl‐2‐oxazolidinethione, molar mass, 179.24 g/mol (right) [Colour figure can be viewed at wileyonlinelibrary.com]

Based on structural similarities and equine administration experiments, barbarin, 5‐phenyl‐2‐oxazolidinethione from *Brassicaceae* plants, is a possible source of aminorex (*R,S*)‐5‐phenyl‐4,5‐dihydro‐1,3‐oxazol‐2‐amine, (Figure 1) identifications in race and sport horse urines. Aminorex is an amphetamine‐related (Hofmaier et al, 2013^1^) US Drug Enforcement Administration (DEA) controlled substance considered illegal in racing and sport horses. Aminorex is also an Association of Racing Commissioners International [ARCI] Class 1, Penalty class A foreign substance, so identifications of aminorex in equine samples can give rise to significant penalties for horsemen (ARCI Uniform Classification Guidelines for Foreign Substances January 2018 (V.13.4),^2^ the suggested penalties being in the order of a 1‐year suspension and a $10,000 fine.

This relationship between plant barbarin and equine urinary aminorex identifications was first suggested by Teale and Biddle (2018),^3^ who had identified aminorex in English sport horse plasma samples with no known exposure to aminorex or levamisole, levamisole being an equine anthelmintic, and immune stimulant known to metabolize to aminorex.^4^, ^5^ Reviewing their aminorex identifications, the absence of any known sources of aminorex, the presence of a number of small plant‐related molecules in their equine plasma samples and the lack of presence of pemoline or rexamino, known metabolites of levamisole,^6^, ^7^ Teale and Biddle^3^ proposed that the likely source of their aminorex identifications was glucobarbarin, a barbarin precursor found in Brassicaceae plants.

**FIGURE 2:**
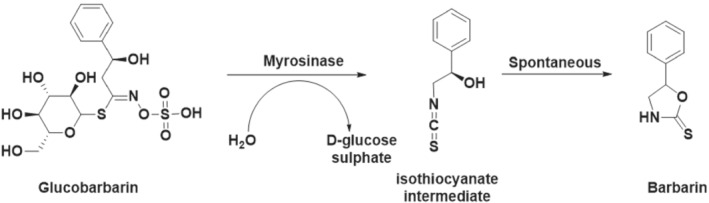
In plants, glucobarbarin is hydrolyzed by the enzyme myrosinase to the isothiocyanate intermediate, above center, which spontaneously cyclizes to barbarin*.*

Plants of the genus *Barbarea Brassicaceae* family contain glucobarbarin, a barbarin precursor. Structural damage in these plants triggers hydrolysis of glucobarbarin by myrosinase to an intermediate which spontaneously cyclizes to barbarin, Figure 2, which then functions as an insect repellant or attractant.^8^ As set forth above, barbarin is related structurally to aminorex, and consumption of *Brassicaceae* plant fragments in equine feed is therefore a possible source of unexplained aminorex identifications, as demonstrated in our recently published research.^9^

While this equine administration research^9^ links consumption of the Brassicaceae plant *Barbarea vulgaris* to urinary aminorex identifications, it does not unequivocally identify barbarin as the proximate chemical source of these identifications. To address this matter, we have streamlined the synthesis, purified, and characterized *d*_5_‐barbarin, the availability of which will allow more definitive identification of the relationship between plant barbarin and equine consumption of such plant material being associated with equine aminorex identifications. *D*_5_‐barbarin was synthesized by a variant of previously described barbarin synthesis methods^10^, ^11^, ^12^, ^13^, ^14^ as follows.

## 2 D_5_‐BARBARIN SYNTHESIS

**FIGURE 3:**
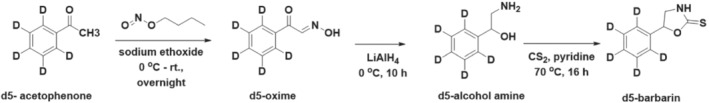
Overall reaction scheme for synthesis of *d*_5_‐barbarin, starting with *d*_5_‐acetophenone, butylnitrite, and sodium ethoxide, yielding *d*_5_‐oxime, followed by reduction with lithium aluminum hydride to give *d*_5_‐phenylethanolamine, which was then reacted with carbon disulfide in the presence of pyridine to yield *d*_5_‐barbarin.

Synthesis of *d*_5_‐barbarin was achieved in three steps, Figure 3 below, a modification of our previously described synthetic methodology. Briefly, *d*_5_‐acetophenone was subjected to a butylnitrite‐mediated transformation to *d*_5_‐2‐oxo‐2‐phenylacetaldehyde oxime (*d*_5_‐oxime) in good yield. Reduction of *d*_5_‐oxime using LiAlH_4_ afforded the hydroxyamine, *d*_5_‐2‐amino‐1‐phenylethan‐1‐ol (*d*_5_ *‐*phenylethanolamine). The final ring closure step to form *d*_5_‐barbarin is an atom‐economy transformation that utilizes carbon disulfide in pyridine as described in our previous reports on the synthesis of unlabeled barbarin.^13^, ^14^ Details on the synthesis are provided in the supporting information.

## 3 EXPERIMENTAL

**FIGURE 4:**
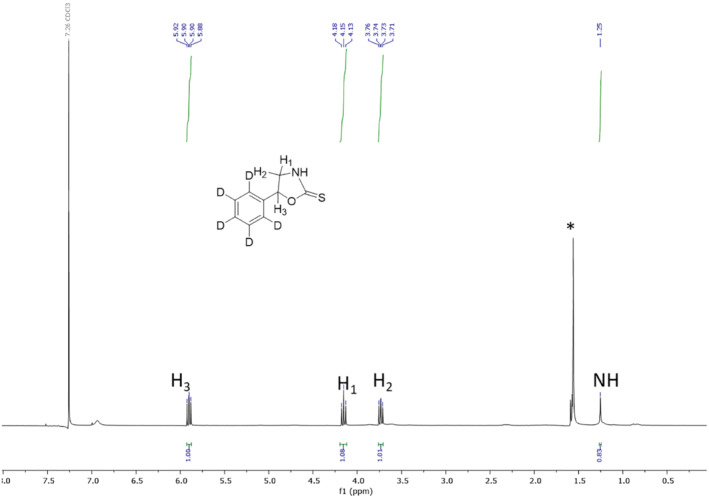
Proton NMR of *d*_5_‐barbarin. * = water [Colour figure can be viewed at wileyonlinelibrary.com]

**FIGURE 5:**
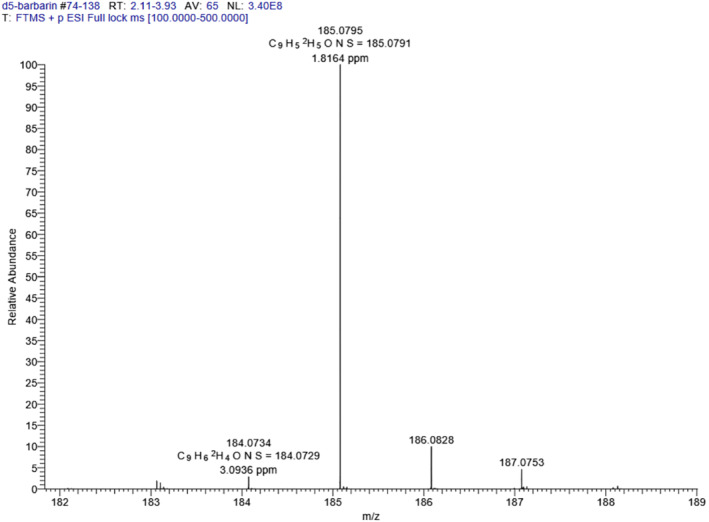
Mass spectrum of *d*5‐barbarin by high‐resolution electrospray ionization‐mass spectrometry [M + H] + for C9H4D5NOS, *m/z* 185.0795, as above, matching an expected value of 185.0791 [Colour figure can be viewed at wileyonlinelibrary.com]

**Synthesis of *d***_**5**_ **‐oxime:** Butyl nitrite (0.96 ml, 8.2 mmol) in ice‐cold ethanol (50 ml) was added to sodium ethoxide (0.565 g, 8.2 mmol) in a reaction vessel. To this solution, *d*_5_‐acetophenone (1 g, 8.0 mmol) dissolved in 10 ml of ethanol was added dropwise over 30 min at 0°C, and the reaction warmed to room temperature overnight. The precipitate formed was filtered, washed with ether, dissolved in a minimum quantity of water acidified with glacial acetic acid, and the resulting off‐white solid filtered and recrystallized from ethanol (0.56 g, 45% yield). The recovered *d*_5_‐oxime material was characterized by ^1^H NMR and mass spectrometry as appropriate for *d*_5_‐oxime, *m/z* 154.0791, ^1^HNMR (DMSO‐*d*_6_, 400 MHz) δ (ppm): ^1^H (s, 8.0, 1H), as in Figure S1 in the supporting information.
**Synthesis of *d***_**5**_ **‐phenylethanolamine:** To a round bottomed flask charged with argon and fitted with a dropping funnel and stir bar was added LiAlH_4_ (0.115 g, 3.04 mmol 4 equivalent) in anhydrous ether (30 ml) and the LiAlH_4_/ether slurry was stirred at 0°C. Then, *d*_5_‐oxime (0.117 g, 0.76 mmol 1 eq), dissolved in anhydrous ether (10 ml), was added dropwise, and the mixture stirred and refluxed for 10 h. Excess hydride was hydrolyzed by water and ether and the white precipitate formed filtered off. The ethereal filtrate was dried over anhydrous Na_2_SO_4_, concentrated to yield a yellow solid (75 mg, 70% yield). The recovered *d*_5_‐phenylethanolamine was characterized by ^1^H NMR (CDCl_3_, 400 MHz) δ (ppm): 4.87 (t,1H), 3.10 (dd, 1H) and 2.85 (dd,1H), 4.80 (1H, OH), 1.98 (2H, NH_2_) as in Figure S2 in the supporting information, HRMS: Found *m/z*: 142.115. Calculated: 142.12.
**Synthesis of *d***_**5**_ **‐barbarin:** *d*_5_‐ phenylethanolamine (0.075 g, 1 eq) in THF (20 ml) was added to excess carbon disulfide (1 ml) in the presence of 1 equivalent of pyridine. The mixture was refluxed at 70°C for 16 h and the reaction monitored by TLC. Upon completion, the reaction was cooled to room temperature, concentrated, and washed with 1 N HCl, water, and the aqueous layer extracted with DCM. The resulting organic layers were combined, dried with Na_2_SO_4_, and the yellow solid recrystallized twice from DCM/hexane solvent system (45 mg, 50% yield). ^1^H NMR (CDCl_3_, 400 MHz) δ (ppm): 5.88 (t, 1H, *J* = 8 Hz), 4.13 (t, 1H, J = 8 Hz), 3.71 (dd, 1H, *J* = 4 Hz, 8 Hz), 1.25 (s, 1H) (Figure 4), and HRMS: Found (*m/z*): [M + H]^+^ for C_9_H_4_D_5_NOS 185.0795. Calculated: 185.0791 (Figure 5). Purity was determined to be >98% by RP‐HPLC: *R*_*f*_ *=* 4.58 min using the following method: Flow rate: 1 ml/min; *λ* = 280 nm; eluent A = DI water with 0.1% trifluoroacetic acid; eluent B = acetonitrile with 0.05% formic acid; solvent gradient: 0–16 min (0:100 H_2_O: ACN), 16 min until end of run (100:0 H_2_O: ACN).

### 3.1 *D*_5_‐barbarin characterization

**FIGURE 6:**
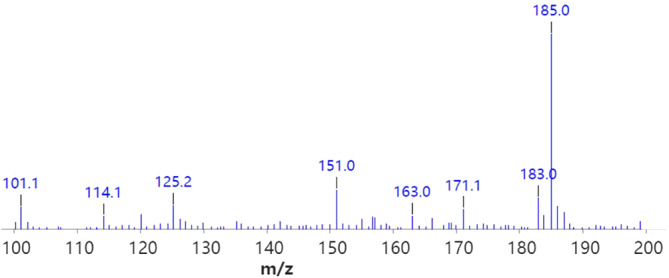
ESI‐MS of *d*5‐barbarin [Colour figure can be viewed at wileyonlinelibrary.com]

The white crystalline material obtained following purification was characterized as follows: Analysis by proton nuclear mass resonance yielded the following, ^1^H NMR (CDCl_3_, 400 MHz) δ (ppm): 5.88 (t, 1H, *J* = 8 Hz), 4.13 (t, 1H, J = 8 Hz), 3.71 (dd, 1H, *J* = 4 Hz, 8 Hz), 1.25 (s, 1H) as shown in Figure 4. High‐resolution mass spectrometry presented [M + H]^+^ for C_9_H_4_D_5_NOS at *m/z* 185.0795, as in Figure 5, and mass spectral product ions (Figure 6) could be interpreted as listed in Table 1. Isotopic abundance analysis also provided excellent agreement with expected *m/*z values and expected relative abundances in high‐resolution mass spectrometry experiments (supporting information Table S2). Based on NMR, HRMS, and HPLC, we have characterized the final product as *d*_5_‐barbarin for use in barbarin‐related research.

**TABLE 1:** Interpretation of *d*_5_‐barbarin ESI‐MS mass spectral product ions

Product ion seen in *d*_5_‐barbarin, *m/z*	Interpretation	Corresponding product ion in *d*_0_‐barbarin, *m/z* ^a^
125	[M + H]^+^ − carbon oxide sulfide (S=C=O)	120
151	[M + H]^+^ − hydrogen sulfide (SH_2_)	146
185	[M + H]^+^	180

*Note*: The corresponding product ions from nondeuterated *d*_0_‐barbarin are listed for comparison.

^a^ Extracted from ESI‐MS of *d*_0_‐barbarin. See Figure S4 in the supporting information.

## 4 DISCUSSION

*D*_5_‐barbarin synthesis commenced with production of *d*_5_‐oxime from *d*_5_‐acetophenone via butylnitrite in an ethoxide/ethanol solution as described by Norman et al. in 1962.^10^ This oxime product was obtained in good yield and reduced with lithium aluminum hydride,^11^ producing the corresponding *d*_5_ *‐*phenylethanolamine, again in good yield. Final ring closure of the *d*_5_ *‐*phenylethanolamine was performed by addition of carbon disulfide in the presence of pyridine, as previously described.^13^, ^14^ The *d*_5_‐barbarin reaction product was presented as a stable white crystalline powder, which was obtained in sufficient yield, purified by recrystallization, and chemically characterized as *d*_5_‐barbarin by proton NMR, HPLC, and ESI‐mass spectrometry and prepared for use as an internal standard in barbarin‐related research.

The research requirement for *d*_5_‐barbarin comes from the apparent ability of *Brassicaceae* plants consumed by horses at pasture in Kentucky, New York, and elsewhere to give rise to low‐concentration urinary identifications of aminorex, a US DEA schedule 1 controlled substance prohibited in racing and sport horses. A previous unexpected source of aminorex identifications was levamisole, a veterinary anthelmintic, and immune stimulant at one time not infrequently prescribed in racing and sport horses. Identification of levamisole administration as a source of aminorex identifications^4^ led to a marked reduction in the number of aminorex identifications in racing horses but not to their complete elimination, as noted by Teale and Biddle in 2018.^3^

## 5 CONCLUSIONS

In closing, *d*_5_‐barbarin has now been synthesized, purified, and characterized. *D*_5_‐barbarin presents as a stable white crystalline substance and is available as a stable isotope internal standard for analytical, forensic, or toxicological research. Additionally, if required, this *d*_5_‐barbarin is available in larger quantities such as may be required for in vitro or in vivo research on the role of barbarin and related plant substances relevant to possible plant sources of aminorex or plant precursors of aminorex giving rise to aminorex identifications in equine drug testing samples.

## Supporting information


**Data S1.** Supporting Information

## Data Availability

The data that support the findings of this study are available from the corresponding author upon reasonable request.
