# Supplementary material for: Synthesis and characterization of d 5‐barbarin for use in barbarin‐related research
Source: Drug Test Anal. 2022 Aug 23;15(1):42–6. doi: 10.1002/dta.3357 (PMC10087961; doi:10.1002/dta.3357)
Supplement: Supplementary file 1 — Data S1. Supporting Information [file DTA-15-42-s001.docx]

**Supplementary Information for**

**SYNTHESIS AND CHARACTERIZATION OF D5-BARBARIN FOR USE IN BARBARIN RELATED RESEARCH**

Sucheta Kudrimoti ^1^¢, Jacob Machin^1^¢, Adedamola S. Arojojoye^2^¢ Samuel G. Awuah^2,3^ Rodney Eisenberg^4^, Clara Fenger^5^, George Maylin^6,^ Andreas F. Lehner^7,^ and Thomas Tobin^1*^

**1/ FIGURE A SUPPLEMENTAL MATERIAL:**

The recovered d_5_-oxime material was characterized by ^1^H NMR and Mass spectrometry as appropriate for d_5-_oxime, *m/z* 154.0791, ^1^HNMR (DMSO-*d_6_*, 400 MHz) δ (ppm): ^1^H (s, 8.0, 1H), as in Figure A supplemental material.

**Fig S1:** The recovered d_5_-oxime material as characterized by ^1^H NMR

**2/ FIGURE B SUPPLEMENTAL MATERIAL:**

The recovered *d_5_-*phenylethanolamine was characterized by ^1^H NMR (CDCl_3_, 400 MHz) δ (ppm): 4.87 (t,1H), 3.10(dd, 1H) and 2.85 (dd,1H), 4.80 (1H, OH), 1.98 (2H, NH_2_) as in Figure B, supplemental material.

**

**Fig S2:** The resultant material was characterized as *d_5_-*phenylethanolamine by ^1^H NMR.

**3/ TABLE 1: SUPPLEMENTAL MATERIAL:**

Table 1. Isotopic mass spectral peak breakdown for the *d_5_*-barbarin [M+H]^+^ complex in comparison to expected *m/z* values calculated with Mass Spec Calculator Pro (version 4.03, Quadtech Associates, 1998). Measured and expected relative percent abundances are also listed.

| **Measured *m/z*** | **Expected *m/z*** | **Measured relative %** | **Expected relative %** |
| --- | --- | --- | --- |
| 185.0795 | 185.0792 | 100 | 100 |
| 186.0826 | 186.0826 | 10 | 10.01 |
| 187.0753 | 187.0750 | 4.56 | 4.43 |

**Fig. S3:** HPLC chromatogram of *d_5_*-barbarin, (λ = 260 nm)


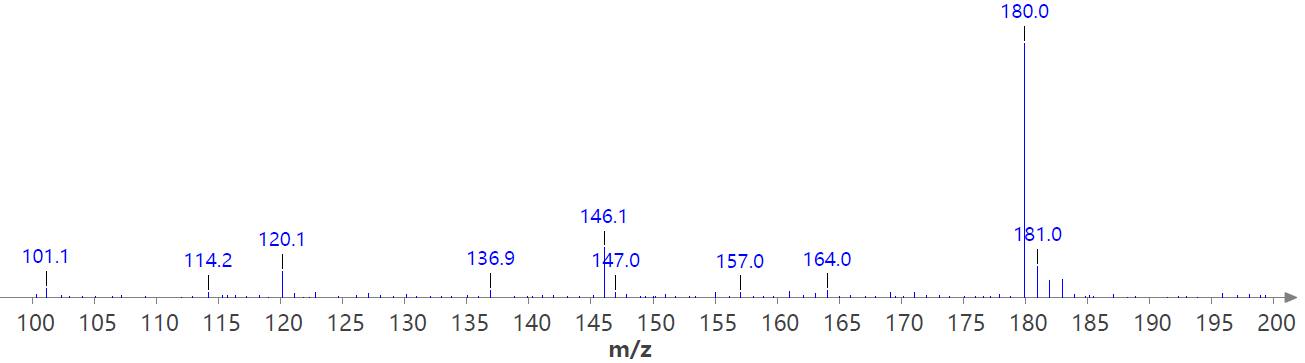


**Fig S4.** ESI-MS of *d_0_*-barbarin
